# Supplementary material for: Oligomeric state of the ZIKV E protein defines protective immune responses
Source: Nat Commun. 2019 Oct 10;10:4606. doi: 10.1038/s41467-019-12677-6 (PMC6787251; doi:10.1038/s41467-019-12677-6)
Supplement: Supplementary file 2 — Reporting Summary [file 41467_2019_12677_MOESM2_ESM.pdf]

nature research

Corresponding author(s): Stefan Metz (NCOMMS-19-18326)
Last updated by author(s): 06/17/2019

Reporting Summary

Nature Research wishes to improve the reproducibility of the work that we publish. This form provides structure for consistency and transparency in reporting. For further information on Nature Research policies, see Authors & Referees and the Editorial Policy Checklist.

Statistics

For all statistical analyses, confirm that the following items are present in the figure legend, table legend, main text, or Methods section.

n/a	Confirmed	
☐	☒	The exact sample size (n) for each experimental group/condition, given as a discrete number and unit of measurement
☐	☒	A statement on whether measurements were taken from distinct samples or whether the same sample was measured repeatedly
☐	☒	The statistical test(s) used AND whether they are one- or two-sided<br>*Only common tests should be described solely by name; describe more complex techniques in the Methods section.*
☒	☐	A description of all covariates tested
☒	☐	A description of any assumptions or corrections, such as tests of normality and adjustment for multiple comparisons
☐	☒	A full description of the statistical parameters including central tendency (e.g. means) or other basic estimates (e.g. regression coefficient) AND variation (e.g. standard deviation) or associated estimates of uncertainty (e.g. confidence intervals)
☒	☐	For null hypothesis testing, the test statistic (e.g. F, t, r) with confidence intervals, effect sizes, degrees of freedom and P value noted<br>*Give P values as exact values whenever suitable.*
☒	☐	For Bayesian analysis, information on the choice of priors and Markov chain Monte Carlo settings
☒	☐	For hierarchical and complex designs, identification of the appropriate level for tests and full reporting of outcomes
☒	☐	Estimates of effect sizes (e.g. Cohen's d, Pearson's r), indicating how they were calculated

Our web collection on statistics for biologists contains articles on many of the points above.

Software and code

Policy information about availability of computer code

Data collection	n/a
Data analysis	Data was graphed and analyzed using GraphPad Prism software.

For manuscripts utilizing custom algorithms or software that are central to the research but not yet described in published literature, software must be made available to editors/reviewers. We strongly encourage code deposition in a community repository (e.g. GitHub). See the Nature Research guidelines for submitting code & software for further information.

Data

Policy information about availability of data

All manuscripts must include a data availability statement. This statement should provide the following information, where applicable:

- Accession codes, unique identifiers, or web links for publicly available datasets
- A list of figures that have associated raw data
- A description of any restrictions on data availability

All data generated or analyzed during this study are included in this published article.

Field-specific reporting

Please select the one below that is the best fit for your research. If you are not sure, read the appropriate sections before making your selection.

☒ Life sciences ☐ Behavioural & social sciences ☐ Ecological, evolutionary & environmental sciences

For a reference copy of the document with all sections, see nature.com/documents/nr-reporting-summary-flat.pdf

Life sciences study design

All studies must disclose on these points even when the disclosure is negative.

Sample size	The sample size (n=5 per group) in the mouse immunogenicity studies has been chosen based on previously published studies where we analyzed DENV subunit immunogenicity.
Data exclusions	No data has been excluded
Replication	All samples have been analyzed in duplicate. Wildtype mouse immunizations has been replicated once in order to generate sufficient sera for passive transfer studies.
Randomization	mice were randomly attributed to experimental groups.
Blinding	Authors were not blinded to group allocation in this study

Reporting for specific materials, systems and methods

We require information from authors about some types of materials, experimental systems and methods used in many studies. Here, indicate whether each material, system or method listed is relevant to your study. If you are not sure if a list item applies to your research, read the appropriate section before selecting a response.

Materials & experimental systems

n/a	Involved in the study	
☐	☒	Antibodies
☐	☒	Eukaryotic cell lines
☒	☐	Palaeontology
☐	☒	Animals and other organisms
☒	☐	Human research participants
☒	☐	Clinical data

Methods

n/a	Involved in the study	
☒	☐	ChIP-seq
☒	☐	Flow cytometry
☒	☐	MRI-based neuroimaging

Antibodies

Antibodies used	4G2, 1M7, C8, C10, A9E, G9E, ZKA-230, 2D22, a-mouse IgG-AP, a-human IgG-AP
Validation	4G2 --> Henchal et al., 1985; Am J Trop Med Hyg<br>1M7 --> Smith et al., 2014; J Vir<br>C8, C10 --> Rouvinsky et al., 2015; Nature<br>A9E, G9E --> Collins et al., 2019; JCI insight<br>ZKA-230 --> Stettler et al., 2016; Science<br>2D22 --> Firbriansah et al., 2015; Science<br>a-Mouse IgG-AP: Sigma (A9316)<br>a-human IgG-AP: Sigma (A1543)

Eukaryotic cell lines

Policy information about cell lines

Cell line source(s)	Vero-81 cell lines. Purchased from ATCC
Authentication	Cell lines used were not authenticated after purchase
Mycoplasma contamination	Cell lines used are mycoplasma free
Commonly misidentified lines (See ICLAC register)	n/a

Animals and other organisms

Policy information about studies involving animals; ARRIVE guidelines recommended for reporting animal research

Laboratory animals	Female C57BL/6 mice were purchased from Jackson Laboratory .<br>Female A129 (B6.129S2-Ifnar1tm1Agt/Mmjax) mice were purchased from MMRRC (Mutant Mouse Resource & Research centers

	supported by NIH)
Wild animals	n/a
Field-collected samples	n/a
Ethics oversight	All mouse experiments were performed under protocols approved by the University of North Carolina Institutional Animal Care and Use Committee, in compliance with federal regulations (the Public Health Service Policy on Humane Care and Use of Laboratory Animals, Animal Welfare Act, and the Guide for the Care and Use of Laboratory Animals).

Note that full information on the approval of the study protocol must also be provided in the manuscript.
